# Dominant Negative FADD/MORT1 Inhibits the Development of Intestinal Intraepithelial Lymphocytes With a Marked Defect on CD8αα+TCRγδ+ T Cells

**DOI:** 10.3389/fimmu.2018.02038

**Published:** 2018-09-10

**Authors:** Xuerui Zhang, Lina Huo, Lulu Song, Zhaoqing Hu, Xinran Wang, Yuheng Han, Ying Wang, Peipei Xu, Jing Zhang, Zi-Chun Hua

**Affiliations:** ^1^The State Key Laboratory of Pharmaceutical Biotechnology, School of Life Sciences, Nanjing University, Nanjing, China; ^2^Changzhou High-Tech Research Institute of Nanjing University and Jiangsu Target Pharma Laboratories Inc., Changzhou, China; ^3^Department of Hematology, Drum Tower Hospital, School of Medicine, Nanjing University, Nanjing, China; ^4^Shenzhen Research Institute of Nanjing University, Shenzhen, China

**Keywords:** FADD, mucosal immune system, γδ+ T-IELs, LPLs, DSS-induced colitis

## Abstract

Intestinal intraepithelial lymphocytes (IELs) play a critical role in mucosal immune system, which differ from thymus-derived cells and develop locally in gut. Although the development of IELs has been studied in some detail, the molecular cues controlling their local development remain unclear. Here, we demonstrate that FADD, a classic adaptor protein required for death-receptor-induced apoptosis, is a critical regulator of the intestinal IEL development. The mice with a dominant negative mutant of FADD (FADD-DN) display an abnormal development of intestinal IELs with a marked reduction in the numbers of CD8αα^+^TCRγδ^+^ T cells. As a precursor for CD8αα^+^ development, lamina propria lymphocytes in lin-negative expression (lin^−^ LPLs) were analyzed and the massive accumulation of IL-7R^−^lin^−^ LPLs was observed in FADD-DN mice. As IL-7R is one of Notch1-target genes, we further observed that the level of Notch1 expression was lower in Lin^−^ LPLs from FADD-DN mice compared with normal mice. The downregulation of Notch1 expression induced by FADD-DN overexpression was also confirmed in Jurkat T cells. Considering that IL-7 and its receptor IL7-R play a differentiation inducing role in the development of intestinal IELs, the influence of FADD via its DD domain on Notch1 expression might be a possible molecular signal involved in the early IELs development. In addition, loss of γδ T-IELs in FADD-DN mice aggravates DSS-induced colitis, suggesting that FADD is a relevant contribution to the field of mucosal immunology and intestinal homeostasis.

## Introduction

Intraepithelial lymphocytes (IELs), which are integral to the intestinal mucosal associated lymphoid system, play a key role in maintaining immune homeostasis of intestine. They constitute a constellation of barrier immune cells and contribute to the intestinal function by developing tolerance to food and microbial antigens in normal physiological state and controlling insults from pathogens and deleterious tissue inflammation during mucosal infections (1, 2). Studies conducted to date have revealed that these T cells consist of two main subpopulations. One bears either CD4 or CD8αβ molecules and TCRαβ, mainly as thymus-dependent. The other bears CD8αα molecules and either TCRγδ or TCRαβ, which is also present in the athymic mice. These thymus-independent IELs like CD8αα^+^ T cells gather mainly in the intestinal mucosa and develop locally (3, 4). The local development tends to facilitate the production of TCRγδ^+^ T cells while it reduces the induction of TCRαβ rearrangements or pre-Tα chain expression. The pre-Tα chain as an essential component of the pre-TCR is responsible for the predominance of TCRαβ production by the thymus (5). Intestinal CD8 T-IELs have been proposed to develop locally from cryptopatch (CP) precursors, whereas the regulatory stages between CP and mature T-IELs remain unclear. There are striking differences in T cell differentiation process in the gut, when compared with T cell differentiation in the thymus, but far less is known about the molecules and signaling pathways that regulate the differentiation.

Fas-associated protein with death domain (FADD) is an adaptor protein critical for the death receptors (DRs) apoptotic signaling (6, 7). When extrinsic apoptosis is triggered, FADD interacts with death receptor (DR) like Fas, leading to the recruitment of procaspase-8 for its activation and then the consequent apoptosis (8–12). Besides being a main death adaptor molecule, FADD participates in other biological processes, such as embryogenesis (9), innate immunity (13), T cell activation and proliferation (14). FADD deficiency leads to inhibition of thymocyte development in a variety of transgenic mouse models including FADD^−/−^ chimeras mice (FADD^−/−^ → RAG-1^−/−^) and T-cell-specific FADD knockout mice (lck-cre FADD) (8, 15–20). In transgenic mice expressing a dominant negative FADD mutant (FADD-DN) under control of the lck promoter, there is also a defect in the progression of thymocytes from CD25^+^CD44^−^ to CD25^−^CD44^−^ phenotype, which is associated with pre-TCR expression. Several studies on FADD-DN transgenic mice have demonstrated its suppression role on T-cell proliferation and supported an acknowledgement of the functional FADD signaling essential for normal T cell development and T cell activation (21–23). But all these studies only focused on TCRαβ^+^ T cells from thymus dependent, this is a meaningful question whether FADD also affects the development of TCRγδ^+^ T cell, such as IELs which are thymus-independent.

Here we identify a novel role for FADD in the intestinal immune system. In FADD-DN transgenic mice, a significant decreased subset of CD8^+^TCRγδ^+^ T cell is observed in intestinal IELs. We provide evidences that FADD-DN expression inhibits the development of CD8^+^TCRγδ^+^ T through impeding the IL-7R expression in their precursors. Loss of CD8^+^TCRγδ^+^ T means an impaired intestinal immunologic barrier, so FADD-DN mice develop more severe inflammation in DSS-induced colitis.

## Results

### Lack of γδ T cells in FADD-DN transgenic mice

**Figure 1:**
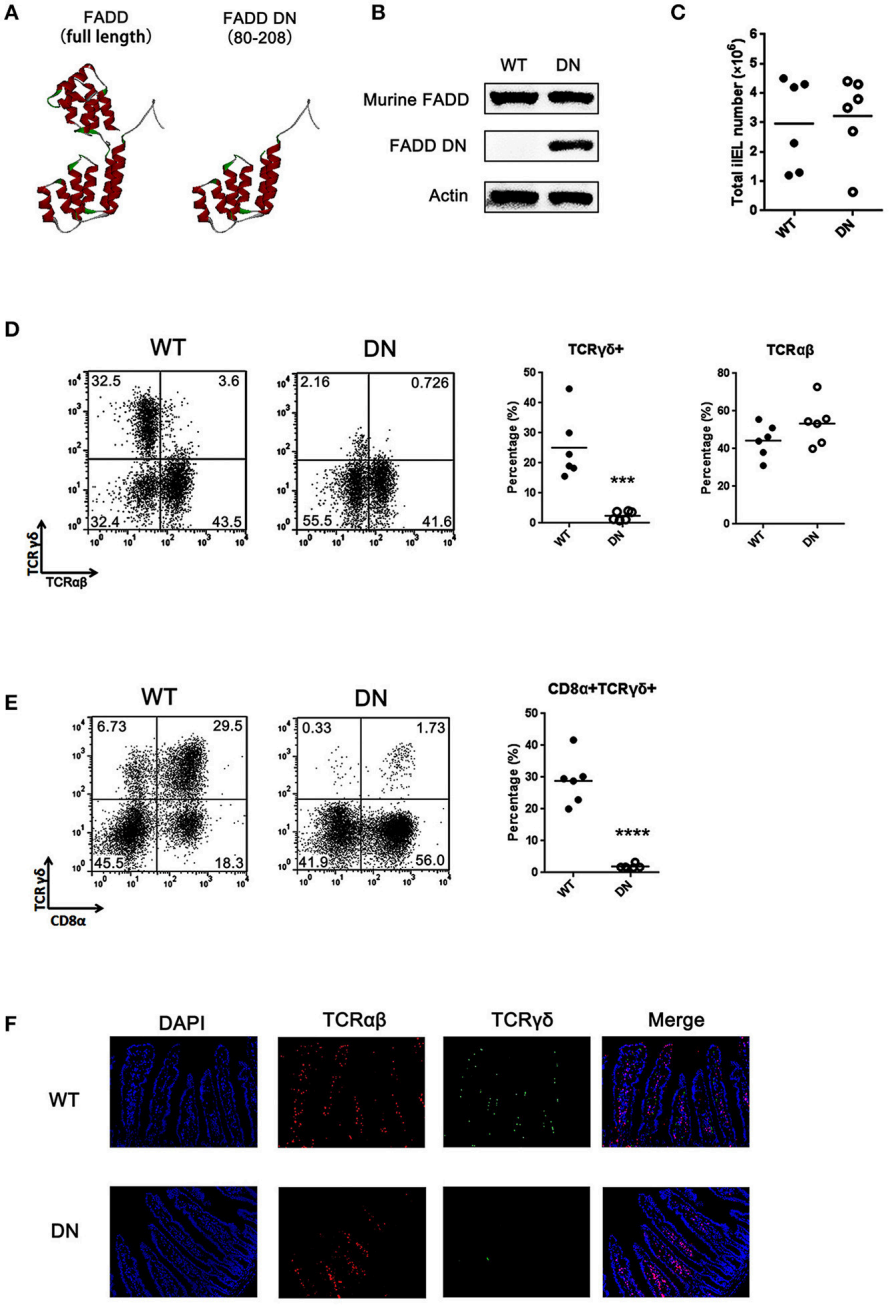
Loss of γδ T cells in FADD-DN transgenic mice. **(A)** The three-dimensional structures of FADD [Protein Data Bank (PDB); accession number: 2GF5] and FADD-DN (death domain of FADD). **(B)** Western blot analysis of FADD expression in mesenteric lymph nodes from wild type (WT) and FADD-DN mice. **(C)** Total IEL numbers of wild type and FADD-DN mice, calculated from six mice per group. Each dot represents one mouse experimental of each group. The solid horizontal lines indicate mean value of each group. **(D)** IELs from WT and FADD-DN mice were stained by Abs to TCRαβ and TCRγδ. The percentage for γδ IELs (TCRγδ^+^ TCRαβ^−^) and αβ IELs (TCRγδ^−^ TCRαβ^+^) were displayed in the appropriate quadrant. Statistic analysis of γδ IELs and αβ IELs from indicated groups (*n* = 6 per group) on the right. Each dot represents one mouse of each experimental group. ***P < 0.001. **(E)** IELs from WT and FADD-DN mice were stained by Abs to CD8α and TCRγδ for FACS analysis. Statistic analysis of CD8α^+^TCRγδ^+^ IELs from indicated groups (n = 6 per group) on the right. Each dot represents one mouse of each experimental group. ****P < 0.0001. **(F)** Immunofluorescent staining of γδ and αβ IELs in intestinal histological sections from WT and FADD-DN mice (*n* > 3 per group). Representative data from at least 3 mice per group is shown.

To test the role of FADD in the development of murine intestinal IELs, the transgenic mice expressing a dominant negative mutant of FADD/MORT1 (FADD-DN) under control of the mouse lck proximal promoter were used in the study. FADD-DN lacks the death effector domain (DED) which is required for recruiting and activating caspase-8 during apoptosis (Figure 1A). A 16 kDa FADD-DN protein in the T-cell specific expression was detected by western blotting (Figure 1B).

Murine intestinal T-IELs are developed by thymic and extrathymic pathways. About half of intestinal T-IELs derives from peripheral lymphoid tissues to the intestine (TCRαβ^+^), and the other half differs from peripheral T cells, mostly expressing TCRγδ^+^ (24). These γδ T-IELs mainly develop locally in the intestinal mucosa. Analysis of intestinal IELs in total number showed no obvious differences in matched wild type (WT) control and FADD-DN mice (Figure 1C). When the subsets of intestinal IELs were examined, TCRγδ^+^ cells were missing in FADD-DN mice (Figures 1D,E). Statistical analysis showed that there were significant differences in TCRγδ^+^ cells or CD8α^+^TCRγδ^+^ cells between WT and FADD-DN mice, while no significant changes in TCRαβ^+^ cells were observed. By immunofluorescence assays in histological sections of the small intestines, the decreased numbers of γδ T-IELs were easier and more direct to be observed (Figure 1F).

### A selective deficiency of CD8αα^+^TCRγδ^+^ T cells caused by FADD-DN expression

**Figure 2:**
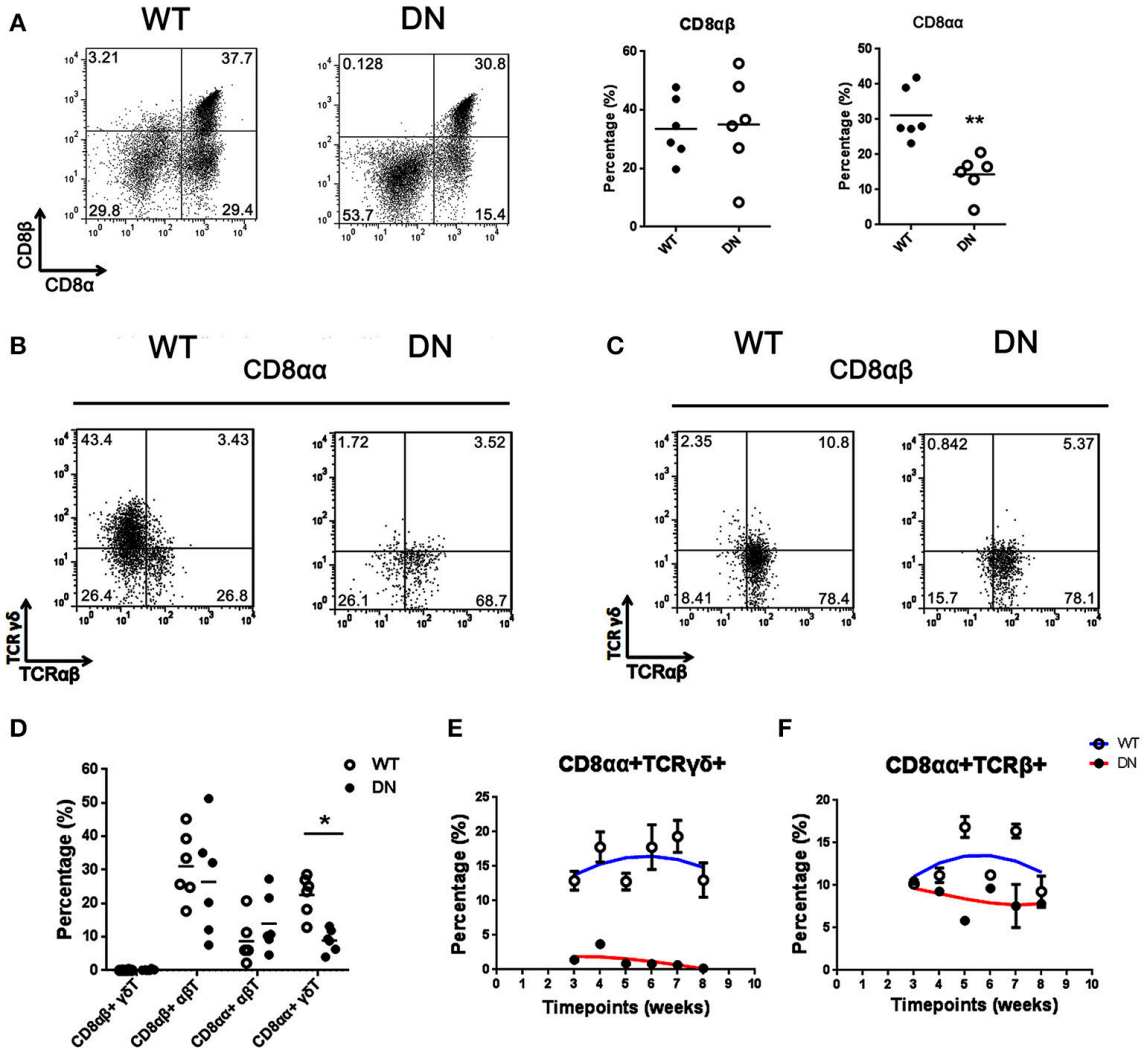
Selective deficiency of CD8αα^+^γδ T cells in FADD-DN mice. **(A)** Representative FACS analysis of CD8αα^+^ (CD8α^+^CD8β^−^) and CD8αβ^+^ (CD8α^+^CD8β^+^) populations in the IELs from WT and FADD-DN mice. Statistical analysis of CD8αα^+^ and CD8αβ^+^IELs from indicated groups (*n* = 6 per group) is on the right. Each dot represents one mouse of each experimental group. **P < 0.01. **(B)** CD8αα^+^ cells gated from **(A)** were analyzed for the expression of TCRαβ and TCRγδ, distinguished into two subsets: CD8αα^+^ αβT (CD8αα^+^TCRαβ^+^TCRγδ^−^) and CD8αα^+^ γδT (CD8αα^+^TCRαβ^−^TCRγδ^+^). **(C)** CD8αβ^+^ cells gated from **(A)** were analyzed for the expression of TCRαβ and TCRγδ, distinguished into two subsets: CD8αβ^+^αβT (CD8αβ^+^TCRαβ^+^TCRγδ^−^) and CD8αβ^+^ γδ T (CD8αβ^+^TCRαβ^−^TCRγδ^+^). **(D)** Statistic analysis of the percentages of indicated subsets among total IELs from WT and FADD-DN mice (*n* = 6 per group). Each dot represents one mouse of each experimental group. *P < 0.05. **(E)** Time-dependent changes of the percentage of CD8αα^+^TCRγδ^+^ subset in the IELs from WT and FADD-DN mice analyzed by FACS. Error bars reflect S.E.M. (*n* = 3 per group). **(F)** Time-dependent changes of the percentage of CD8αα^+^TCRβ^+^ subset in the IELs from WT and FADD-DN mice analyzed by FACS. Error bars reflect S.E.M. (*n* = 3 per group). Data was calculated from three mice per group.

T-IELs consist mainly of two populations of CD8^+^ T cells. One bears CD8αβ^+^TCRαβ^+^; the other bears homodimeric α/α CD8 chains with TCRγδ^+^ or TCRαβ^+^, and it is mainly thymo-independent (25). Two subgroups of CD8αα^+^ and CD8αβ^+^ were gated respectively for testing the proportional changes of TCRγδ^+^ or TCRαβ^+^. The percentage of CD8αα^+^ IELs from FADD-DN mice was significantly reduced compared with WT mice, while no significant differences were shown in CD8αβ^+^ IELs (Figure 2A). In CD8αα^+^ subset, over 40% of CD8αα^+^ T expressing TCRγδ were observed in WT mice, but few γδ T cells were observed in FADD-DN mice (Figure 2B). The population of αβ T in CD8αβ^+^ subset showed no obvious changes in both mice (Figure 2C). By comparison of statistic analysis, the effect of FADD-DN was mainly observed on the depletion of CD8αα^+^ γδ T-IELs (Figure 2D). The properties of the IELs exhibit age-related changes. The total number of IELs are gradually increased until 8 weeks old and the development of CD8αα^+^ T cell subsets also tends to be stable at 8 weeks, both in the γδ and αβ lineages (26, 27). So we analyzed a group of FADD-DN and littermate control mice aged 3–8 weeks old and found that CD8αα^+^TCRγδ^+^ cells always maintained low number along the time-span in FADD-DN mice, and a similar kinetic on CD8αα^+^TCRαβ^+^ cells occurred in both mice (Figures 2E,F). These results suggest that FADD-DN expression inhibits the development of CD8αα^+^ γδ T cells.

### The effect of FADD-DN on T-IELs development is thymo-independent

**Figure 3:**
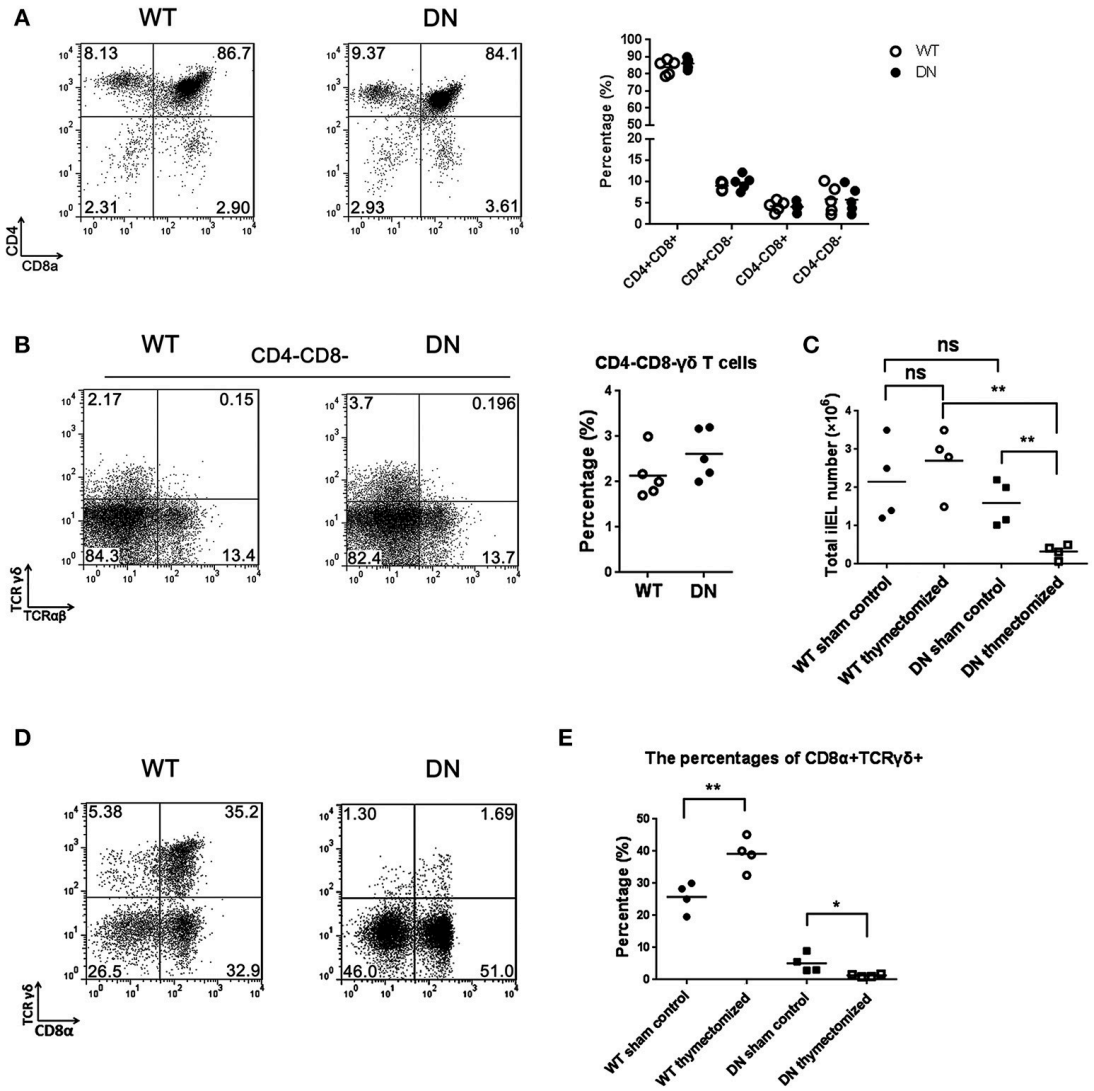
The effect of FADD-DN on intestinal IELs development. **(A)** Representative FACS analysis of thymus from wild type and FADD-DN mice (*n* = 5 per group). Thymocytes were stained by Abs to CD4 and CD8. Statistical analysis of the subsets of CD4^+^CD8^+^, CD4^+^CD8^−^, CD4^−^CD8^+^, and CD4^−^CD8^−^ thymocytes from indicated groups is shown on the right. Each dot represents one mouse of each experimental group. **(B)** CD4^−^CD8^−^ T cells of thymus were gated from **(A)** and then analyzed for the markers of TCRγδ and TCRαβ. Representative FACS analysis on the left and statistical analysis of percentages of CD4^−^CD8^−^ γδ T cells (CD4^−^CD8^−^ TCRαβ^−^TCRγδ^+^) on the right (*n* = 5 per group). Each dot represents one mouse of each experimental group. **(C)** Total IELs numbers of WT and FADD-DN mice before or after thymectomy. The changes of total IEL numbers between control and thymecomized mice are shown in the statistical analysis (*n* = 4 per group). Each dot represents one mouse of each experimental group. **(D)** IELs from WT and FADD-DN mice were obtained 4 weeks after thymectomy. Flow cytometry was performed to analyze the percentage of CD8α^+^TCRγδ^+^ populations stained by Abs to CD8α and TCRγδ. **(E)** The percentages of CD8α^+^TCRγδ^+^ subset among total IELs from indicated group(*n* = 4 per group). Each dot represents one mouse of each experimental group. *P < 0.05; **P < 0.01.

Intestinal T-IELs may originate from both thymus and extrathymus (24). To distinguish the pathway for FADD-DN to regulate the development of γδ T cells, we examined the percentage of CD4^−^CD8^−^ γδ T cells in the thymus by FACS. There were no significant differences in the thymic cellularity between FADD-DN mice and WT mice (Figure 3A). In the subsets of CD4^−^CD8^−^ T cells, the percentage of γδ T cells from FADD-DN mice was also similar to WT mice (Figure 3B), indicating that the development of γδ T cells in the thymus was not defective in FADD-DN mice.

In euthymic mice, there are some competitions for local cytokines between thymic αβ T cells and extrathymic T cell progenitors, so the extrathymic development of T-IELs is severely repressed (28). In the athymic mice, the extrathymic lymphopoiesis emerges with a priority toward γδ T cells, which localizes mainly in the intestinal mucosa leading to the accumulation of T-IELs (29, 30). To confirm the effect of FADD-DN on the local development of intestinal IELs, the thymectomy was performed on both WT and FADD-DN mice. After 4 weeks, analysis of total intestinal IEL numbers in thymectomized mice showed a marked reduction (about 6-fold) in FADD-DN mice (Figure 3C), suggesting that there was a defect on IEL development. By comparison of the proportion of CD8α^+^TCRγδ^+^ T cells before or after thymectomy, more serious reductions of CD8α^+^TCRγδ^+^ T were observed in FADD-DN mice with thymectomy (Figures 3D,E). Thus, it is reasonable to speculate that the local γδ T-IELs are deficient in FADD-DN mice, and the few intestinal γδ T-IELs before thymectomy seem to mainly derive from thymus originally. Taken together, the deficiency of γδ T-IELs caused by FADD-DN might be a sign of stunting local development.

### The development of IELs is arrested at stage of Lin^−^ LPLs in FADD-DN mice

Murine intestinal IELs originate from their own pre-existing stem cells present in the intestine (i.e., cryptopatches) (4, 31, 32) and appendix (33). Intestinal T cell precursors have specific phenotype: lineage markers negative (Lin^−^) Thy^+^c-kit^+^IL-7R^+^CD44^+^CD25^+^ (4). To determine at which point the differentiation of IELs was plagued by FADD-DN, we examined the Lin^−^ cells prepared from intestinal IELs and lamina propria lymphocytes (LPLs). A mixture of mAbs was prepared to exclude mature cell types and isolate Lin^−^ cells (see section Materials and Methods).

**Figure 4:**
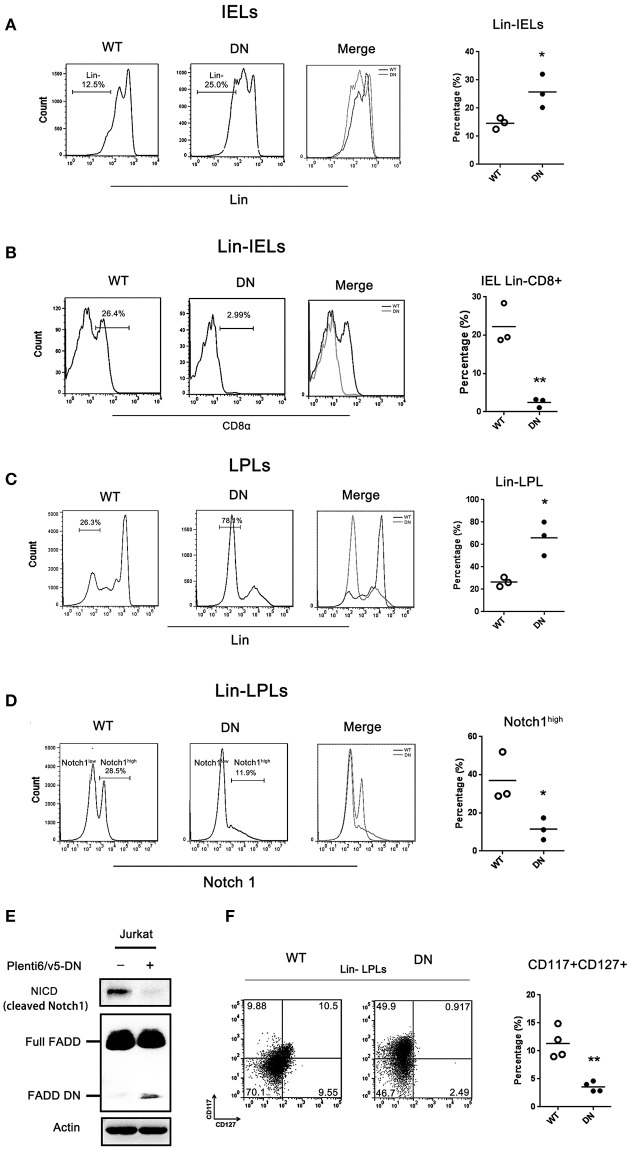
The development of IELs arrested by FADD-DN in Lin^−^ LPLs with IL-7R deficiency. **(A)** IELs from WT and FADD-DN mice were labeled with a mixture of mAbs recognizing mature hematopoietic cells to gate Lin^−^ populations as described in methods. FACS plots show the gating strategy for the population of Lin^−^ IELs. Statistic analysis of Lin^−^ IELs from indicated groups (*n* = 3 per group) is shown on the right. Each dot represents one mouse of each experimental group. *P < 0.05. **(B)** The Lin^−^ IELs gated from **(A)** were labeled with antibody to CD8α. Histograms show the expression level of CD8α in Lin^−^ IELs from indicated mice. Statistic analysis of CD8α^+^ subset in Lin^−^ IEL from indicated groups (*n* = 3 per group) is shown on the right, Each dot represents one mouse of each experimental group. **P < 0.01. **(C)** LPLs from WT and FADD-DN mice were stained with antibodies against lineage markers (Lin).FACS plots show the gating strategy for Lin^−^ LPL populations. Statistic analysis for Lin^−^ LPLs on the right (*n* = 3 per group). Each dot represents one mouse of each experimental group. *P < 0.05. **(D)** The Lin^−^ LPLs gated from **(C)** were labeled with antibody to Notch1. Histograms show two levels of Notch1 expression in Lin^−^ LPLs indicated as Notch1^high^ and Notch1^low^. Statistic analysis for Notch1^high^ Lin^−^ LPLs on the right (*n* = 3 per group). Each dot represents one mouse of each experimental group. *P < 0.05. **(E)** Jurkat T cell were transiently transfected with FADD-DN expression vector. The level of NICD (cleaved Notch1) was detected by western blot. **(F)**The Lin^−^ LPLs gated from **(C)** were labeled with anti-CD117and anti-CD127 Abs. Note: CD117 as c-kit and CD127 as IL-7R. Statistical analysis of the percentages of CD127^+^ LPLs on the right (*n* = 4 per group), Each dot represents one mouse of each experimental group. **P < 0.01.

By FACS analysis, there was about 25% Lin^−^ IELs in FADD-DN mice and about 12.5% in WT mice, and a significant difference between two groups (Figure 4A). Gating on Lin^−^ IELs for further analysis of CD8α expression, the percentage of Lin^−^CD8^+^ IELs was dramatically decreased in FADD-DN mice (Figure 4B), suggesting that the differentiation of intestinal T-IELs might be blocked in Lin^−^ CD8^−^ stage. For a better understanding of intestinal T-IEL maturation in earlier events, we examined Lin^−^ LPLs which have been confirmed equal to precursors Lin^−^ CP. A massive accumulation of Lin^−^ LPLs was observed in FADD-DN mice, which is a clear hint on the attested stage of intestinal T-IEL development (Figure 4C). Our previous research found that FADD deficiency inhibits thymocyte development at the β-selection checkpoint by modulating Notch1 signaling (8). Since Notch1 is essential for T differentiation and specifying the cell fate, the expression of Notch1 in progenitor Lin^−^ LPLs was detected. Notably, there were two different groups of Notch1 expression: high expression as Notch1^high^ and low expression as Notch1^low^ in Lin^−^ LPLs from WT mice, but in FADD-DN mice, both groups of Notch1^high^ and Notch1^low^ were totally different from WT mice (Figure 4D). The proportion of Notch1^high^ Lin^−^ LPLs was a noticeable decrease in FADD-DN mice. To reconfirmed the effect of FADD-DN on Notch1 expression, FADD-DN expression vector was transfected into Jurkat T cells. Consistent with *in vivo* study, overexpression of FADD-DN in Jurkat T cells also led to a significant decrease in Notch1 expression (Figure 4E).

IL-7R is one of Notch1-target genes and Notch1 controls T cell development in part by regulating the stage- and lineage-specific expression of IL-7R (34, 35). C-Kit is a tyrosine kinase receptor and extrathymus-derived IELs normally in older mice are c-Kit-dependent (4, 36). These two crucial markers: CD117 (c-kit) and CD127 (IL-7R) can be used to distinguish a lineage of T cells with unique developmental attributes. By comparisons of the subsets in Lin^−^ LPLs marked with CD117 and CD127, there was a distinct difference in CD127^+^ (IL-7R expression) between WT mice and FADD-DN mice (Figure 4F). In FADD-DN mice, Lin^−^ LPL shared little expression of surface antigen IL-7R, just in line with the low expression of Notch1. The enterocyte-produced IL-7 plays a differentiation inducing role in the development of intestinal IELs (37, 38). In order to fully develop, the thymic-independent TCRγδ^+^ IELs in an immature state must interact with their appropriate ligands *in situ*, so lack of IL-7R in Lin^−^ LPL cells from FADD-DN mice might provide a novel role for FADD in early intestinal T-IEL development.

### The role of FADD-DN in intestinal immunoregulation

**Figure 5:**
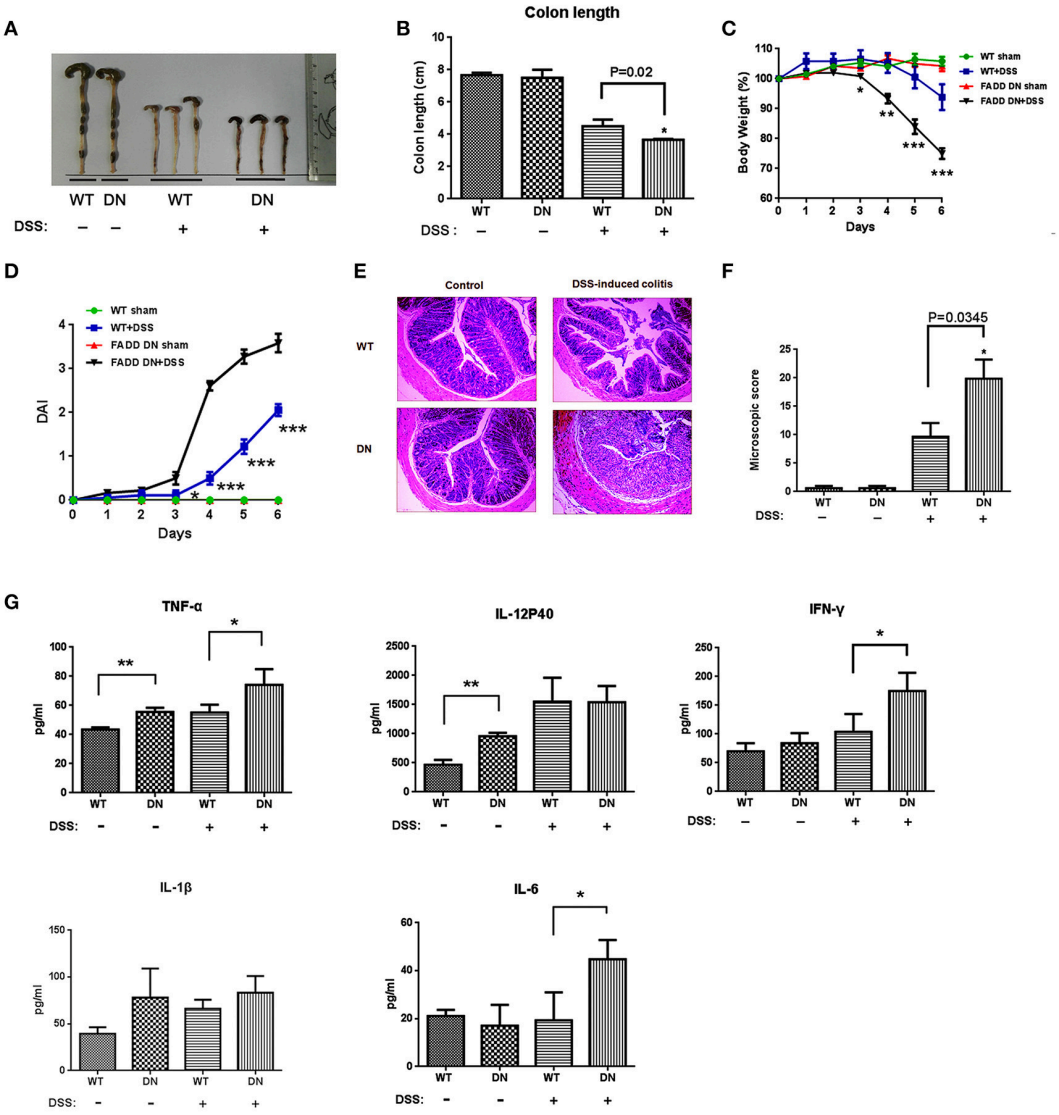
More severe DSS-induced colitis in FADD-DN mice. Mice (*n* = 6 per group) were given 3% DSS in their drinking water for 5 days and then provided with water for 1 day before being sacrificed. Macroscopic appearances **(A)** and colon lengths **(B)** were measured in indicated groups. **(C)** Body weights of mice in each group (*n* = 6 per group) were recorded daily. The changes of body weights induced by DSS-induced colitis were shown in the percentage of the original body weight. **(D)** Disease activity index (DAI) was calculated as described in the material and methods section. **(E)** Colon sections of indicated groups with H&E staining. The original amplification was 100×. **(F)** Histological scores of colon sections were calculated as described in the Materials and methods. **(G)** Serum levels of cytokines in different groups detected by ELISA. Data shown here are from a representative experiment repeated three times with similar results. *P < 0.05, **P < 0.01, ***P < 0.001 vs. DSS-treated vehicle group.

As the first-line defense against infectious agents, γδ T-IELs are involved in intestinal immunoregulation (39). Depletion or deficiency in γδ T-IELs aggravates intestinal inflammation in almost every investigated model, especially DSS-induced colitis (40). Our finding about loss of γδ T-IELs in FADD-DN mice draws substantial interest in its pathological responses. DSS-induced colitis model was established in both WT and FADD-DN mice. Severe illness, which was characterized by diarrhea, intestinal bleeding, body weight loss, and shortened colon length, was observed at 4 days after DSS administration. Compared with WT group, FADD-DN mice exhibited severe colitis with a distinct reduction in colon length (Figures 5A,B). From day 3 to day 6 of DSS-induced colitis, a significant loss of body weight was observed in FADD-DN mice compared to WT mice (Figure 5C), and the disease activity index (DAI) of FADD-DN mice also increased more dramatically (Figure 5D). The pathological section with H&E staining showed more severe pathological changes in DSS-treated FADD-DN mice, including loss of goblet cells, distortion of crypts, mucosal damage and necrosis (Figure 5E). Correspondingly, histological score of colon sections was much higher in FADD-DN mice (Figure 5F). Consistent with these phenotypes, higher levels of TNF-α, IFN-γ, and IL-6 were detected in the serum of FADD-DN mice, while no significant differences in IL-12p40 and IL-1β (Figure 5G). It is worth mentioning that a higher level of several cytokines (TNF-α, IL-12p40, and IL-1β) was detected in sham group of FADD-DN mice, suggesting FADD-DN in the T-cell specific expression might affect basic immune response.

## Discussion

To date, a plethora of reports has clearly identified FADD as an essential adaptor between death receptors and caspase-8 (41, 42). Non-apoptotic functions of FADD have been implicated in T lymphocyte development and activation, mostly in thymic αβ T cells. To clarify the role of FADD in the development of γδ T cells, FADD-DN mice provide a useful tool to dissect T cell development in the mucosa for thymic and extrathymic pathways. Based on our findings, FADD plays an important role in intestinal IEL development, especially in γδ T-IELs which are thought to develop locally and largely via an extrathymic pathway.

T-IELs play a critical role in regulating intestinal mucosal immune responses. γδ T-IELs are a crucial protective T cell subset against colitis (43). Loss of intestinal γδ T-IELs in FADD-DN mice is a main phenotype. The decreased CD8α^+^TCRγδ^+^ T cells will take responsibility for the reduced homeostasis in intestinal mucosal immune system, so FADD-DN mice show more severe inflammation in DSS-induced colitis model (Figure 5). To track the differentiation of intestinal CD8α^+^TCRγδ^+^ T cells, we prepared intestinal T cell precursors referring to Lambolez's paper published on J. Exp. Med. (4). The Lin^−^ LPLs (or namely Lin^−^ WL) isolated by the reporting method has been fully demonstrated to be cryptopatch (CP) precursors. These Lin^−^ LPLs have specific phenotype: (Lin^−^) Thy^+^c-kit^+^IL-7R^+^CD44^+^CD25^+^. Analysis of Lin^−^ IELs and Lin^−^ LPLs revealed an arrest for CD8α^+^TCRγδ^+^ T development at stage of IL-7R^−^c-kit^+^Lin^−^ LPLs in FADD-DN mice. The IL-7R is expressed on lymphoid T and B precursors, and innate lymphoid cells. Its ligand IL-7 is integral to T and B cell development in primary lymphoid organs, so IL-7/IL-7R plays an essential role in supporting T cell development and homeostasis. The loss of IL-7R expression in intestinal precursors might be responsible for the defect on intestinal T-IEL development in FADD-DN mice, because the local development of IELs is IL-7-dependent. The possible molecular mechanism involved in the deficiency of IL-7R expression in Lin^−^ LPLs is the decreased Notch1 expression induced by FADD-DN overexpression (Figure 4).

We have reported previously that FADD regulates thymocyte development at the β-selection checkpoint by modulating Notch1 signaling (8). Notch1 mRNA increases from early thymocyte progenitor to the DN3a stage and markedly decreases at the DN3b stage, commensurate with pre-TCR signaling. Differential expression of Notch1 represents a distinct lineage of T cells with unique developmental and functional attributes (44). Consistent with the reports, we also have detected two states of Notch1 expression in intestinal T-IEL precursors, but the regulation for Notch1 expression is suppressed by FADD-DN expression (Figure 4). These results suggest that FADD is a part of signals for promoting intestinal T-IEL development through its DD domain. Of course, the signal that is inhibited by FADD-DN may or may not come from a DD-receptor signaling. It is possible that there are other binding molecules with FADD yet to be discovered. NKAP known as a Notch1 transcriptional repressor was previously identified by us to associate with FADD, implying a potential role of FADD in maintaining the stabilization of NKAP to inhibit Notch1 on transcriptional level. Besides being cytoplasmic, the FADD protein is also localized in the nucleus of many types of cells and nuclear FADD is probably implicated in a functional transcription factor complex to modulate Notch1 expression (8). The study of FADD function on intestine IEL development by Notch1 still needs further investigation, which will give a comprehensive understanding of FADD function in the field of mucosal immunology.

In summary, the present study demonstrates a novel function of FADD in the development of intestinal T-IELs, especially on CD8α^+^TCRγδ^+^ population. Several important immune functions have been reported for intestinal γδ T cells, including the first-line defense against pathogens and an immuno-down regulatory role during infection (45, 46). γδ T cell-deficient mice spontaneously develop colitis at 8 weeks of age (46). Decreased numbers of γδ T cells are observed in areas of Crohn's colitis (47) and depletion of γδ T cells aggravates TNBS colitis resulting in significant mortality (40, 48). Further investigation of FADD on mucosal immunology seems meaningful since the influence on CD8α^+^TCRγδ^+^ subset mediated by FADD might be involved in the initiation and/or perpetuation of colitis.

## Materials and methods

### Cell culture

Jurkat T cells (ATCC, Shanghai, China) were maintained in RPMI 1640 (Gibco-BRL, Basel, Switzerland) containing antibiotics and 10% FCS (Gibco-BRL). Human embryonic kidney (HEK)-293 T adherent cells (ATCC, Shanghai, China) were cultured in DMEM (Gibco-BRL) supplemented with antibiotics and 10% FCS (Gibco-BRL).

### Mice

FADD DN mice were provided by the laboratory of Aster Winoto (University of California, Berkeley), which are generated and maintained on C57BL/6J background. Mice C57BL/6J were purchased from Beijing Animal Centre, and maintained in pathogen-free conditions. All animal experiments were approved by Nanjing University Animal Care and Use Committee, and we strictly followed the recommendation of the guidelines of the Animal Care Committee of Nanjing University.

### Isolation of intraepithelial lymphocytes

Isolation of intestinal Intraepithelial lymphocytes (IELs) was performed as described previously (2). After washing the small intestine to remove fecal content, Peyer's patches and fat tissue were extirpated. The small intestine was opened longitudinally, cut into small pieces, and washed with ice-cold Ca^2+^- and Mg^2+^-free CMF-HBSS buffer containing 1 mM dithiothreitol (Sigma) three times. The intestines were then incubated with CMF-HBSS supplemented with penicillin, streptomycin, and 10% FCS and shaken at 220 rpm, 37°C. After filtrating the supernatants through a nylon mesh, IELs were collected by a 44–67% Percoll density gradient (Solarbio, Beijing, China) layered between the 44–67%. After washed by PBS, cells were counted and used for the following assays.

### Isolation of lamina propria lymphocytes (LPL)

After IELs isolation, tissues were digested in RPMI1640 supplemented with 200 U/ml collagenase type XI (Sigma, USA), 0.1 mg/ml DNase I (invitrogen), and 10% FCS at 220 rpm, 37°C for 60 min. Lamina propria lymphocytes (LPLs) were released and then isolated with 44–67% Percoll fractionation as described above.

### Dextran sodium sulfate (DSS)-induced colitis

FADD DN or wild-type mice were fed with water charged with 3.0 % (W/V) DSS (dextran sulfate sodium salt, 36–50 kDa, 0216011080, MP Biomedicals) for 5 days and then with normal drinking water for 1 day till sacrificed. Mice given normal water served as control. Body weight of mice was recorded daily. Mice were sacrificed 6 days after DSS exposure. The length of the colons for each group were measured. After washing by PBS, colons tissue were cut and fixed in 4% (v/v) formaldehyde for histological analysis.

### Evaluation of disease activity

During the experiment, body weight of mice for each group was recorded daily and feces were collected. Disease activity index(DAI) was determined by loss of weight, stool consistency, and fecal blood. The calculation of DAI was performed as previously (49).

### Histological analysis

The colons were fixed in formaldehyde, embedded in paraffin and cut into serial sections of 3-μm-thick for histological analysis. Stained with haematoxylin and eosin (H&E), tissues were analyzed by histological grading according to the criteria described previously (50).

### Immunofluorescence assay

The deparaffinized colon tissues underwent antigen retrieval and were blocked with 10% goat serum. Sections were incubated with anti-mouse TCRαβ and TCRγδ primary antibodies (1:50, Cell Signaling Technology (CST), Danvers, MA, USA) overnight at 4°C. After washing with PBS three times for 5 min, sections were incubated with appropriate secondary antibodies (1:1000; invitrogen) at room temperature for 30 min. Following washed with PBS three times for 5 min, slides were counterstained with DAPI (invitrogen). All images of the slides were visualized and captured by fluorescence microscope (Zeiss AX10, Carl Zeiss AG, Germany).

### Construction and transfection of FADD-DN lentiviral vector

The sequence of FADD-DN was cloned into a plenti6/v5-D-Topo expression vector (Invitrogen) using restriction enzyme BamHI and XhoI. Primers for amplifying the DNA sequence of FADD-DN: forward: 5′-CGGGATCCATGGACGACTTCGA-3′ and reverse: 5′-CCCTCGAGTCAGGACGCTTCGGAGGT-3′. Briefly, HEK-293T cells were co-tranfected with FADD-DN expression plasmid and lentiviral envelope plasmids (PL3, PL4, and PL5). The viruses were harvested by ultra-centrifugation on day 3 after transfection.

Jurkat T cells was replaced with transduction medium. 8 μg/ml polybrene (Santa Cruz Biotechnology) was added followed by lentiviral transduction. Virus was removed 24 h after transduction and 10 μg/ml blasticidin was added for another 48 h. The cells were cultured for 72 h to examine the expression of FADD-DN by western blot.

### Western blotting assay

Samples of protein lysates were separated by 10% SDS-PAGE and electro-transferred to PVDF membrane. After blocking with 5% non-fat milk for 2 h at room temperature, membranes were incubated with antibodies against mouse FADD (ab124812; Abcam, Cambridge, UK), human FADD C-terminus (610399; BD Pharmingen, Franklin Lake, NJ, USA), β-actin (AM1021B; Abgent, San Diego, CA, USA), and cleaved Notch1 (4147s; Cell Signaling Technology, Danvers, MA, USA) followed by suitable secondary antibody conjugated with horseradish peroxidase (Jackson Immunoresearch Laboratories, West Grove, PA, USA). Reactive bands were detected with enhanced chemiluminescence solution (Tanon, Shanghai, China) and visualized with an imaging program (Tanon, Shanghai, China).

### Cytokine measurement

Cytokines in serum samples including TNF-α, IL-6, IL-17, IL-1β, IL-12 were measured using ELISA kits (R&D Systems, US) according to the manufacturer's instructions.

### Flow cytometry analysis

The IELs were stained with antibodies against lineage markers (Lin) as follows: anti-CD3 (145-2C11), anti-CD19 (1D3), anti-TCRαβ (H57-597), anti-TCRγδ (GL3), anti-erythroid cells (TER-119), anti-GR1 (8C5), anti–CD11b (M170), anti-IgM (LL41), and anti-CD8b (eBioH35-17.2). All the antibodies for FACS were purchased from BD Pharmingen (San Diego, CA). CD8αα cells were gated as CD8α^+^CD8β^−^. Fluorescence was measured with a flow cytometer (FACS Calibur; BD Biosciences) equipped with Cell Quest software (BD Biosciences, Canada).

### Thymectomy

Thymectomy was performed on 8-week-old mice as described before (32). A ventilator (V-100; Yuyan instruments, Shanghai, China) was used to keep mice breathing during the operation. Completeness of thymectomy was confirmed by visual inspection, both directly after removal of the organ and at the end of the experiment. Only fully thymectomized animals were included in this study.

### Statistical analysis

The experimental data were presented as mean ± SEM. The statistical significance of the differences between groups was evaluated by One-way ANOVA and Student's *t*-test (*P* < 0.05).

## Author contributions

XZ and LH conceptualized the study, designed and carried out the experiments. XZ wrote the manuscript. LS, ZhH, and XW participated and analyzed the experiments. YH, YW and PX provided many materials and discussed the results. JZ and ZiH revised the manuscript and provided important advices. XZ and LH contributed equally to this work.

### Conflict of interest statement

The authors declare that the research was conducted in the absence of any commercial or financial relationships that could be construed as a potential conflict of interest. The reviewer DS and handling Editor declared their shared affiliation.

## References

[1] Cheroutre H Lambolez F Mucida D. The light and dark sides of intestinal intraepithelial lymphocytes. Nat Rev Immunol. (2011) 11:445–56. 10.1038/nri3007 21681197 PMC3140792

[2] Wang X Sumida H Cyster JG. GPR18 is required for a normal CD8αα intestinal intraepithelial lymphocyte compartment. J Exp Med. (2014) 211:2351–9. 10.1084/jem.20140646 25348153 PMC4235638

[3] Ettersperger J Montcuquet N Malamut G Guegan N Lopezlastra S Gayraud S. Interleukin 15-dependent T cell-like innate intraepithelial *Lymphocytes* develop in the intestine and transform into *Lymphomas* in celiac disease. Immunity (2016) 45:610–25. 10.1016/j.immuni.2016.07.018 27612641

[4] Lambolez F Azogui O Joret AM Garcia C von Boehmer H Di Santo J. Characterization of T cell differentiation in the murine gut. J Exp Med. (2002) 195:437–49. 10.1084/jem.20010798 11854357 PMC2193617

[5] Mallis RJ Bai K Arthanari H Hussey RE Handley M Li Z. Pre-TCR ligand binding impacts thymocyte development before αβTCR expression. Proc Natl Acad Sci USA. (2015) 112:8373–8. 10.1073/pnas.1504971112 26056289 PMC4500245

[6] Lu JV Weist BM van Raam BJ Marro BS Nguyen LV Srinivas P. Complementary roles of Fas-associated death domain (FADD) and receptor interacting protein kinase-3 (RIPK3) in T-cell homeostasis and antiviral immunity. Proc Natl Acad Sci USA. (2011) 108:15312–7. 10.1073/pnas.1102779108 21876153 PMC3174674

[7] Dowling JP Nair A Zhang J. A novel function of RIP1 in postnatal development and immune homeostasis by protecting against RIP3-dependent necroptosis and FADD-mediated apoptosis. Front Cell Dev Biol. (2015) 3:12. 10.3389/fcell.2015.00012 25767797 PMC4341114

[8] Zhang X Dong X Wang H Li J Yang B Zhang J. FADD regulates thymocyte development at the β-selection checkpoint by modulating Notch signaling. Cell Death Dis. (2014) 5:e1273. 10.1038/cddis.2014.198 24901044 PMC4611708

[9] Zhang H Zhou X Mcquade T Li J Chan FK Zhang J. Functional complementation between FADD and RIP1 in embryos and lymphocytes. Nature (2011) 471:373–6. 10.1038/nature09878 21368761 PMC3072026

[10] Kikuchi M Kuroki S Kayama M Sakaguchi S Lee KK Yonehara S. Protease activity of procaspase-8 is essential for cell survival by inhibiting both apoptotic and nonapoptotic cell death dependent on receptor-interacting protein kinase 1 (RIP1) and RIP3. J Biol Chem. (2012) 287:41165. 10.1074/jbc.M112.419747 23071110 PMC3510816

[11] Pennarun B Meijer A de Vries EG Kleibeuker JH Kruyt F de Jong S. Playing the DISC: turning on TRAIL death receptor-mediated apoptosis in cancer. Biochimica et Biophysica Acta (2010) 1805:123–40. 10.1016/j.bbcan.2009.11.004 19961901

[12] Grunert M Gottschalk K Kapahnke J Gündisch S Kieser A Jeremias I. The adaptor protein FADD and the initiator caspase-8 mediate activation of NF-κB by TRAIL. Cell Death Dis. (2012) 3:e414. 10.1038/cddis.2012.154 23096115 PMC3481141

[13] Bonnet MC Preukschat D Welz PS Van LG Ermolaeva MA Bloch W. The adaptor protein FADD protects epidermal keratinocytes from necroptosis *in vivo* and prevents skin inflammation. Immunity (2011) 35:572–82. 10.1016/j.immuni.2011.08.014 22000287

[14] Bell BD Leverrier S Weist BM Newton RH Arechiga AF Luhrs KA. FADD and caspase-8 control the outcome of autophagic signaling in proliferating T cells. Autophagy (2008) 105:16677–82. 10.1073/pnas.0808597105 18946037 PMC2575479

[15] Zhang JK Cado D Chen A Kabra NH Winoto A. Fas-mediated apoptosis and activation-induced T-cell proliferation are defective in mice lacking FADD/Mort1. Nature (1998) 392:296–300. 10.1038/32681 9521326

[16] Newton K Harris AW Strasser A. FADD/MORT1 regulates the pre-TCR checkpoint and can function as a tumour suppressor. Embo J. (2000) 19:931–41. 10.1093/emboj/19.5.931 10698935 PMC305633

[17] Zhang J Kabra NH Cado D Kang C Winoto A. FADD-deficient T cells exhibit a disaccord in regulation of the cell cycle machinery. J Biol Chem. (2001) 276:29815–18. 10.1074/jbc.M103838200 11390402

[18] Kabra NH Kang CH Hsing LC Zhang JK Winoto A. T cell-specific FADD-deficient mice: FADD is required for early T cell development. P Natl Acad Sci USA. (2001) 98:6307–12. 10.1073/pnas.111158698 11353862 PMC33464

[19] Zhang YH Rosenberg S Wang HM Imtiyaz HZ Hou YJ Zhang JK. Conditional fas-associated death domain protein (FADD): GFP knockout mice reveal FADD is dispensable in thymic development but essential in peripheral T cell homeostasis. J Immunol. (2005) 175:3033–44. 10.4049/jimmunol.175.5.3033 16116191 PMC3110086

[20] Osborn SL Diehl G Han SJ Xue L Kurd N Hsieh K. Fas-associated death domain (FADD) is a negative regulator of T-cell receptor-mediated necroptosis. P Natl Acad Sci USA. (2010) 107:13034–9. 10.1073/pnas.1005997107 20615958 PMC2919948

[21] Newton K Harris AW Bath ML Smith KG Strasser A. A dominant interfering mutant of FADD/MORT1 enhances deletion of autoreactive thymocytes and inhibits proliferation of mature T lymphocytes. Embo J. (1998) 17:706–18. 10.1093/emboj/17.3.706 9450996 PMC1170420

[22] Newton K Kurts C Harris AW Strasser A. Effects of a dominant interfering mutant of FADD on signal transduction in activated T cells. Curr Biol. (2001) 11:273–6. 10.1016/S0960-9822(01)00067-7 11250157

[23] Walsh CM Wen BG Chinnaiyan AM O'Rourke K Dixit VM Hedrick SM. A role for FADD in T cell activation and development. Immunity (1998) 8:439–49. 10.1016/S1074-7613(00)80549-X 9586634

[24] Peaudecerf L dos Santos PR Boudil A Ezine S Pardigon N Rocha B. The role of the gut as a primary lymphoid organ: CD8Î±Î± intraepithelial T lymphocytes in euthymic mice derive from very immature CD44+ thymocyte precursors. Mucosal Immunol. (2011) 4:93–101. 10.1038/mi.2010.47 20737000

[25] Wurbel MA Malissen M Guygrand D Malissen B Campbell JJ. Impaired accumulation of antigen-specific CD8 *Lymphocytes* in chemokine CCL25-deficient intestinal epithelium and lamina propria. J Immunol. (2007) 178:7598–606. 10.4049/jimmunol.178.12.7598 17548595 PMC2564614

[26] Helgeland L Brandtzaeg P Rolstad B Vaage JT. Sequential development of intraepithelial gamma delta and alpha beta T lymphocytes expressing CD8 alpha beta in neonatal rat intestine: requirement for the thymus. Immunology (1997) 92 447–56. 10.1046/j.1365-2567.1997.00379.x 9497485 PMC1364149

[27] Suzuki H Jeong KI Doi K. Age-related changes in the regional variations in the number and subsets of intraepithelial lymphocytes in mouse small intestine. Dev Comp Immunol. (2002) 26:589–95. 10.1016/S0145-305X(02)00004-6 12031418

[28] Jameson SC. Maintaining the norm: T-cell homeostasis. Nat Rev Immunol. (2002) 2:547–56. 10.1038/nri853 12154374

[29] Delphine GG Orly A Susanna C Sylvie D Nussenzweig MC Philippe K Extrathymic T Cell *Lymphopoiesis*. J Exp Med. (2003) 197:333–41. 10.1084/jem.20021639 12566417 PMC2193840

[30] Guy-Grand D Vassalli P. Gut intraepithelial lymphocyte development. Curr Opin Immunol. (2002) 14:255–9. 10.1016/S0952-7915(02)00330-8 11869901

[31] Saran N Łyszkiewicz M Pommerencke J Witzlau K Vakilzadeh R Ballmaier M. Multiple extrathymic precursors contribute to T-cell development with different kinetics. Blood (2010) 115:1137–44. 10.1182/blood-2009-07-230821 20009033 PMC2826228

[32] Herbrand H Pabst O Cryptopatches and Isolated Lymphoid Follicles: aspects of development, homeostasis and function. Dev Biol Peripheral Lymphoid Organs (2011) 107–17. 10.1007/978-3-642-14429-5_10

[33] Ford MS Zhang ZX Chen W Zhang L Double-negative T regulatory cells can develop outside the thymus and do not mature from CD8+ T cell precursors. J Immunol. (2006) 177:2803–9. 10.4049/jimmunol.177.5.2803 16920915

[34] Laky K Lefrancois L von Freeden-Jeffry U Murray R Puddington L. The role of IL-7 in thymic and extrathymic development of TCR gamma delta cells. J Immunol. (1998) 161:707–13. 9670946

[35] Laky K Lefrancois L Ishikawa H Olson S Suzuki K Ishimaru K Enterocyte expression of IL-7 induces development of TCR gamma delta cells and Peyer's patches. Faseb J. (1999) 13:A622.

[36] Saito H Kanamori Y Takemori T Nariuchi H Kubota E Takahashi-Iwanaga H. Generation of intestinal T cells from progenitors residing in gut cryptopatches. Science (1998) 280:275–8. 10.1126/science.280.5361.275 9535655

[37] Shitara S Hara T Liang B Wagatsuma K Zuklys S Holländer GA. IL-7 produced by thymic epithelial cells plays a major role in the development of thymocytes and TCRγδ+ intraepithelial lymphocytes. J Immunol. (2013) 190:6173–9. 10.4049/jimmunol.1202573 23686483

[38] Cai YJ Wang WS Liang HY Sun LH Teitelbaum DH Yang H. Keratinocyte growth factor up-regulates Interleukin-7 expression following intestinal ischemia/reperfusion *in vitro* and *in vivo*. Int J Clin Exp Pathol. (2012) 5:569–580. 22949940 PMC3430112

[39] Ismail AS Severson KM Vaishnava S Behrendt CL Yu X Benjamin JL. Gammadelta intraepithelial lymphocytes are essential mediators of host-microbial homeostasis at the intestinal mucosal surface. Proc Natl Acad Sci USA. (2011) 108:8743–8. 10.1073/pnas.1019574108 21555560 PMC3102410

[40] Kuhl AA Pawlowski NN Grollich K Loddenkemper C Zeitz M Hoffmann JC. Aggravation of intestinal inflammation by depletion/deficiency of gammadelta T cells in different types of IBD animal models. J Leukocyte Biol. (2007) 81:168–75. 10.1189/jlb.1105696 17041003

[41] Nogusa S Thapa RJ Dillon CP Liedmann S Oguin TH III Ingram JP. RIPK3 activates parallel pathways of MLKL-driven necroptosis and FADD-mediated apoptosis to protect against influenza A virus. Cell Host Microbe (2016) 20:13–24. 10.1016/j.chom.2016.05.011 27321907 PMC5026823

[42] Wang PX Ji YX Zhang XJ Zhao LP Yan ZZ Zhang P Targeting CASP8 and FADD-like apoptosis regulator ameliorates nonalcoholic steatohepatitis in mice and nonhuman primates. Nat Med. (2017) 23:439–49. 10.1038/nm.4290 28218919

[43] Meehan TF Witherden DA Kim CH Sendaydiego K Ye I Garijo O. Protection against colitis by CD100-dependent modulation of intraepithelial γδ T lymphocyte function. Mucosal Immunol. (2013) 7:134–42. 10.1038/mi.2013.32 23695512 PMC3795871

[44] Ciofani M Zuniga-Pflucker JC. Notch promotes survival of pre-T cells at the beta-selection checkpoint by regulating cellular metabolism. Nat Immunol. (2005) 6:881–8. 10.1038/ni1234 16056227

[45] Chen YP Chou K Fuchs E Havran WL Boismenu R. Protection of the intestinal mucosa by intraepithelial gamma delta T cells. P Natl Acad Sci USA. (2002) 99:14338–43. 10.1073/pnas.212290499 12376619 PMC137885

[46] Inagaki-Ohara K Chinen T Matsuzaki G Sasaki A Sakamoto Y Hiromatsu K. Mucosal T cells bearing TCRgammadelta play a protective role in intestinal inflammation. J Immunol. (2004) 173:1390–8. 10.4049/jimmunol.173.2.1390 15240735

[47] Nanno M Shiohara T Yamamoto H Kawakami K Ishikawa H. γδ T cells: firefighters or fire boosters in the front lines of inflammatory responses. Immunol Rev. (2007) 215:103–13. 10.1111/j.1600-065X.2006.00474.x 17291282

[48] Hoffmann JC Peters K Henschke S Herrmann B Pfister K Westermann J Role of T lymphocytes in rat 2,4,6-trinitrobenzene sulphonic acid (TNBS) induced colitis: increased mortality after gamma delta T cell depletion and no effect of alpha beta T cell depletion. Gut (2001) 48:489–95. 10.1136/gut.48.4.489 11247892 PMC1728226

[49] Saiz ML Cibrian D Ramírezhuesca M Torralba D Morenogonzalo O Sánchezmadrid F. Tetraspanin CD9 limits mucosal healing in experimental colitis. Front Immunol. (2017) 8:1854. 10.3389/fimmu.2017.01854 29312336 PMC5742144

[50] Zhang X Wei L Wang J Qin Z Wang J Lu Y. Suppression colitis and colitis-associated colon cancer by anti-S100a9 antibody in mice. Front Immunol. (2017) 8:1774. 10.3389/fimmu.2017.01774 29326691 PMC5733461

